# Nurse rostering with fatigue modelling: Incorporating a validated sleep model with biological variations in nurse rostering

**DOI:** 10.1007/s10729-022-09613-4

**Published:** 2022-10-05

**Authors:** Kjartan Kastet Klyve, Ilankaikone Senthooran, Mark Wallace

**Affiliations:** 1 Department of Industrial Economics and Technology Management, Norwegian University of Science and Technology, Trondheim, Norway; 2 Monash University, Melbourne, Australia

**Keywords:** Nurse rostering, Fatigue, Sleep, Mixed-Integer programming, Constraint programming, Large neighbourhood search, Operations research

## Abstract

We use a real Nurse Rostering Problem and a validated model of human sleep to formulate the Nurse Rostering Problem with Fatigue. The fatigue modelling includes individual biologies, thus enabling personalised schedules for every nurse. We create an approximation of the sleep model in the form of a look-up table, enabling its incorporation into nurse rostering. The problem is solved using an algorithm that combines Mixed-Integer Programming and Constraint Programming with a Large Neighbourhood Search. A post-processing algorithm deals with errors, to produce feasible rosters minimising global fatigue. The results demonstrate the realism of protecting nurses from highly fatiguing schedules and ensuring the alertness of staff. We further demonstrate how minimally increased staffing levels enable lower fatigue, and find evidence to suggest biological complementarity among staff can be used to reduce fatigue. We also demonstrate how tailoring shifts to nurses’ biology reduces the overall fatigue of the team, which means managers must grapple with the issue of fairness in rostering.

## Introduction

Adverse psychological and physiological effects of night rotations on nurses are well documented [45]. Impaired vigilance and performance occurs as a result of increased sleepiness and can seriously compromise workers’ health and safety [7], as well as patient safety [31, 36, 66] and [12]. This underscores the importance of avoiding nurse rosters that cause fatigue. Shift work regulations have been established to hinder employee exhaustion. Such rules and regulations are a key part of the constraints in the Nurse Rostering Problem (NRP), see for example [10]. Because such rules over-simplify the conditions underlying fatigue, sleep deprivation and different kinds of fatigue continue to have adverse effects on nurses.

In this work we expand from the typical NRPs to incorporate modeling of fatigue using a validated sleep model. We include individual biology in the fatigue modelling and minimise fatigue to enhance nurse health and reduce the risk of human errors due to impaired vigilance. This is formalised in the Nurse Rostering Problem with Fatigue (NRPwF). We incorporate an approximation of the [49] fatigue model in the form of a lookup-table. The NRPwF is used to solve realistic problem instances based on real-life data. Our research demonstrates that the worst cases of fatigue can be significantly reduced. It serves as a proof of concept for incorporating a general sleep model in an NRP, and is generalisable to other rostering and workforce planning problems. The NRPwF implementation produces rosters minimising the global maximum fatigue, and demonstrates how biology is an important factor when creating fatigue minimising rosters. We further demonstrate how minimally increasing the number of staff makes it possible to significantly reduce the fatigue experienced by nurses.

Our main contributions are listed below:

- Creating an approximation of an advanced sleep model, and demonstrating it can be integrated into the novel Nurse Rostering Problem with Fatigue (NRPwF)
- Introducing realistic biological profiles enabling personalised schedules
- Studying the concept of fairness in rostering in light of insights into individual fatigue
- Creating a new algorithm combining Mixed-Integer Programming and Constraint Programming to facilitate a Large Neighbourhood Search solving the NRPwF, with a post-processing procedure to handle cases where the approximation is erroneous
- Demonstrating how minimising maximum cases of fatigue affect rostering depending on biology, and how increased staffing levels reduce fatigue

The outline of this paper is as follows. In Sections 2.1, 2.2, and 2.3 we present relevant literature in sleep research and nurse rostering. In Section 3, the fatigue model at the core of our project is presented, and preliminary analyses of its effects are performed. We go on to create a typical NRPwF in Section 4, and demonstrate how a fatigue model approximation can be utilized despite cases of imprecision. This is done by implementing an algorithm to find high quality solutions, that are based on the approximation, in Section 5. In Section 6 the use of our algorithm is demonstrated, verified, and in some cases post-processed as part of our computational study. We further perform analyses on the effects of rosters in light of biological profiles and staffing levels. In Section 7, we make concluding remarks and give suggestions for future research.

## Related literature

This section introduces literature on typical NRPs and the most prominently used solution methods. It briefly reviews how fatigue is included in Operations Research (OR) literature, as well as presenting relevant fatigue models from the realm of sleep research. Lastly it summarises the identified gaps in related literature.

### Key concepts of nurse rostering literature

The NRP is a scheduling problem which assigns a number of shifts with predefined start and end times to a set of nurses in a given planning period.

NRPs typically include coverage constraints, ensuring a minimum number of nurses on duty in each shift; time related constraints e.g. a number of hours to be worked by each nurse during the planning period; and constraints capturing work regulations [10].

A range of different rules and regulations exist in the nurse rostering literature. The many different variations are too many to mention explicitly, but scheduling rules that ensure nurses have sufficient rest times are of particular relevance in this work.

Examples of works that avoid double shifts and too many consecutive work days are [24] and [35]. For additional details on the NRP we refer readers to [10, 29], and [13] for an overview of the problem type and to [16] and [62] for two recent examples of papers on nurse rostering.

In this work, as no widely accepted standard NRP exists, we formulate an NRP based on guidelines from Safe Work Australia [61].

Additionally, some more ambiguous concepts complicate the modelling of some NRPs. One important concept is fairness. While there is no agreed definition of fairness in the NRP literature, authors frequently claim to model it. Typically, this entails an even distribution of something considered desirable or undesirable. [26] states “In OR the definition of fairness is vague. It usually takes the form of preventing inequalities”.

This statement concurs with the work of [60], which calculates a score for preference fulfilment for each nurse and maximizes the lowest score. Similar approaches are presented in [65], which investigate fairness in NRPs specifically, and propose measures that balance out soft constraint violations. Similarly, [4] balances out the number of granted requests by using a piecewise linear function of penalties increasing with the number of unfulfilled requests for a particular nurse. Different interpretations of fairness also exist in the literature, e.g. the hierarchical understanding of fairness in [2].

However, assuming fairness in NRPs is mostly understood as an even distribution of something desirable or undesirable, a question of philosophical nature should be posed; does this even distribution reflect a desire for equality or equity among staff? That is, when literature on nurse rostering e.g. balances out night shifts, is this done because an equal number of night shifts (equality) is considered a truly fair distribution, or is it simply the best proxy for equity in fatigue? Consequently, if equity is fully or partly relevant for the fair distribution of fatiguing shifts, then a tool for estimating individual staff fatigue incorporated in the rostering process will improve fairness in rosters. Until now this discussion has only been of theoretical value as no tool to evaluate the individual fatigue of rosters has existed. However, in this work we define fairness as a measure of equity in fatigue, and optimise to find the most fair rosters. Simply put: we create rosters with (as near as possible) equally tired staff rather than balancing out shifts that are assumed tiring.

A second important concept affected by the availability of our fatigue model is patient safety. While the relation between fatigue due to sleep deprivation and performance is subject to ongoing research, models combining homeostatic and circadian drives (such as our fatigue model) can be used to predict a variety of performance and sleepiness measures [25, 54]. The reduction in performance accelerates with increased fatigue levels [57]. Similarly to our equity-focused definition of fairness, this motivates the optimisation of rosters to find the lowest possible worst case of fatigue.

### Solution methods applied to nurse rostering

NRPs are solved in numerous ways, e.g. Artificial Intelligence (AI) approaches, Constraint Programming (CP), metaheuristics and mathematical programming approaches [22]. In the realm of CP, [21] tackles nurse rostering, among other problems, with lazy clause generation. Other examples of CP include [50] and [55]. Examples of AI methods include [30], which builds on CP and integrates fuzzy constraints with branch and bound. The hybrid artificial bee colony algorithm presented in [5] is another AI method used, where the bee operator is replaced with the hill climbing optimizer. According to [11], metaheuristic methods seem to be the dominant technique when solving real-world problems. Examples are the combined bat algorithm and particle swarm optimization by [14], ant colony optimisation with semi random initialization by [1] the tabu search based metaheuristic of [60], and the case-based reasoning approach of [28].

There are three main drawbacks to these meta-heuristic approaches. Firstly they have parameters that require tuning for each application. Secondly, once the parameters have been tuned for a certain set of example inputs, it is unclear for which other inputs the same tuning works. Thirdly the user has no feedback if the parameter tuning is incorrect. The results may be good, or poor, but unless there is another approach to compare them with the user cannot know.

In the realm of mathematical programming approaches, the standard Mixed-Integer Programming (MIP) models are among the most explored ones, see e.g. [4, 6], and [44]. Different decomposition methods have also been explored, with variants of column generation being popular modeling choices [19, 37], and [71]. These approaches typically provide optimality gaps, which give some confidence about the solution quality.

Notably, the literature on nurse rostering often focuses on solution techniques [47]. We argue there should be an increased focus on creating models that are useful in practice and that provide insights for real-life decision makers.

In particular, we have found no published research on nurse rostering integrating a validated sleep model.

We address this gap, in Section 5, by designing an NRP that focuses on minimising fatigue to reduce risks of accidents and improve nurse health, and create managerial insights for decision makers based on our computational results. This implies less focus on proof of technical concepts such as the optimality gap. Rather, we focus on:

- finding high-quality solutions in terms of reducing fatigue more than the standard scheduling rules do within reasonable run times for realistic instances, and
- identifying managerial insights.

### Fatigue modeling literature

When models in OR deal with subjects such as tiredness, stress and work strain, they often present some version of a fatigue model. The term *fatigue* is used ambiguously. It is often loosely defined, if defined at all. In [42] a job rotation tool designed to provide less monotonous and repetitive tasks for employees is presented. Authors define fatigue as “the physical stress that each process induces on the operators”, and it was shown in [43] that job rotation plans could reduce the total accumulated physical fatigue per operator. According to [34], “Fatigue is a stochastic factor that changes according to other factors such as environmental conditions, work type, and work duration”, which they handle using chance constraints. In [27], fatigue is not defined explicitly, but rather linked to road transport crashes and falling asleep while driving, in an effort to evaluate regulations.

While the different approaches to modelling fatigue in the examples mentioned above are useful, literature in medical sciences and biology often distinguishes between acute fatigue and chronic fatigue, further differentiated into muscular fatigue, mental fatigue, psychomotor fatigue and chronic fatigue associated with post-viral syndromes [18]. We argue that literature within sleep research best fits the fatigue experienced by shift workers. This literature deals with fatigue fitting the definition provided in [18]: “the drive to sleep”. This is the sense in which we use the term *fatigue* in this work.

There are several models of human sleep that can be utilized either directly or as part of quantitative tools to evaluate the fatigue of shift workers, e.g. [3, 8, 32, 40, 41, 53, 56, 67]. These models tend to be used as tools for retrospective evaluation. It is rare for such tools to be deployed prospectively, i.e. explicitly incorporating them into models that perform planning. However, we have identified some few examples of this.

The fatigue model in [73] is based on the [32] “Sleep, Activity, Fatigue, and Task Effectiveness (SAFTE)” model, and incorporates it in a staff scheduling tool, where the SAFTE-model has been used to simulate the fatigue score of all possible schedules and ranking them in four categories depending on degree of fatigue. In [75], the authors were inspired by the Fatigue Audit Inter Dyne (FAID) system [59]. [75] simplified the FAID model, resulting in a linearisation of an exponential function suitable for a MIP framework. The problem considers minimising fatigue in work shift scheduling for air traffic controllers. The linear fatigue model is improved in [76], by the addition of a dampening parameter in cases of extreme fatigue. This is shown to fit the results of the FAID model better. A similar model and technique is used for shift scheduling of aircraft maintenance crews in [39]. [38] present a MIP model for nurse scheduling taking into account fatigue using two different approaches. The first is survey-based and the second uses a sinusoidal function that includes a parameter implying individual nurses’ chronotype (propensity to sleep at different times), based on work presented in [17] to approximate fatigue at the end of a week.

[9] proposes the TDSPFM, a Truck Driver Scheduling Model where fatigue is modeled using the non-linear fatigue model proposed in the model of [33], which is itself based on the three process model of [3]. The non-linear TDSPFM is solved using the evolutionary algorithm of the built-in Excel 2013 solver.

Our fatigue modelling is based on the work of [49], where a validated model of human sleep and circadian rhythms is presented. It is discussed in more detail in Section 3. We aim to minimise the changes and adjustments to it, both to conserve the realism provided by the sleep model itself and to ensure the continued relevance of our approach as sleep models are improved and extended. We utilise an approximation technique unused in related literature; namely a lookup table. This technique, presented in Sections 3.2 and 3.3 is conceptually simple, but the generality of the approximation technique makes it relevant when sleep research progresses and new models are produced, as long as the implicit assumption holds.

The referenced works based on validated sleep models have made significant adjustments to models to fit the OR-framework. Our general approximation technique is augmented with post-processing, as described in Section 6.2.1, to find solutions that truly match the validated sleep model.

All works where sleep models are used in prospective planning, except [38], assume homogeneous biology among staff. Furthermore, a review of fatigue (which includes works dealing with fatigue related to sleep drive) in personnel scheduling and operations was published recently. It states: “In conclusion, we view the ‘next frontier’ of work on this topic as being the development of fatigue prediction, measurement and mitigation models within operational research that are calibrated for individual workers. Although challenging, this is a direction that has potential for great research opportunities and practical benefits.” [77] In this work, we take individual nurse biology into account in the prospective planning of fatigue minimising rosters, leading to interesting managerial insights, described in Sections 6.3 and 6.4.

## The fatigue model

In this section we present a fatigue model based on the sleep model of [49]. This sleep model has been subject to testing and parameter-tuning, and similar models have been based on it since. It combines the [48] model of the ascending arousal system with the [23] human circadian pacemaker. In the fatigue model, a sleep/wake switch is included, which models how a human falls asleep and wakes up as a result of internal processes in the brain and light conditions. The impact of shift work on the model is that it precludes sleep. During the times a person is at work, the fatigue model is restricted from entering a sleeping state. While there is a large set of parameter values defined in the sleep model of [49], such as parameters related to internal brain processes and predefined times for dusk and dawn, these values are not altered in the fatigue model and are considered fixed in this work. Only two inputs are provided by us to the fatigue model: biological profiles (discussed in Section 3.1) and the time of forced wakefulness (work and commuting time).

This functionality of forced wakefulness has been used in other works, such as [48] and [25] to model total sleep deprivation, [51] to model shift work, and [64] and [72] to model work schedules. The fatigue model of [49] is written in Matlab [20] and solved using a built-in ordinary differential equation solver.

A notable characteristic of typical rostering problems, as opposed to more general scheduling problems, is that a set of possible shifts is defined. Our NRPwF model admits four shifts in accordance with [61] guidelines for managing the risk of fatigue at work to represent realistic and advisable shift times:

- Day shift “D” 07:00 - 15:00
- Evening shift “E’ 14:30 - 22:30
- Night shift “N” 22:00 - 07:30(+1 day)
- Off-shift “O”

To add to the realism, we have chosen to include 45 minutes of forced wake-time before and after work, to represent commuting. Depending on the roster a nurse works, the fatigue model calculates fatigue based on his or her shifts.

The initial values of the fatigue model variables reflect a well-rested individual where the circadian rhythm has been given time to stabilize in the individual’s preferred phase. To ensure this, we simply let the sleep model run for long periods without any work, thus obtaining the default initial fatigue model state. The individual has typical biological parameter values, meaning default parameter values from the model of [49] are used. These have been validated in previous works. For details see [48, 49], and [23].

**Fig. 1:**
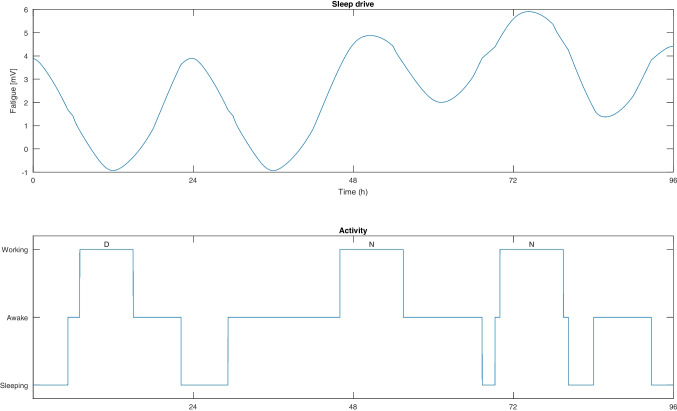
Plots of how fatigue and activity of a nurse with typical biological parameters change as time passes. The nurse is scheduled to work the 4-day roster {D,N,N,O}

In Fig. 1, two plots of a four day example roster {D,N,N,O} are presented. The fatigue is illustrated in the top plot and visibly oscillates according to the time of the day (hours 0, 24, etc. represent midnight). As a result of the two night shifts, the fatigue level is notably higher during the third and fourth day, compared to the two previous days. The sleep drive typically exists in an interval [-2mV,8mV]^1^ depending on biological parameters and other factors that affect sleep. In this work, it is sufficient to compare fatigue values knowing that a lower fatigue is always beneficiary. However, for a more intuitive understanding of how different periods of sleep deprivation correspond to different values of fatigue, see e.g. [25].

In the bottom plot of Fig. 1, the activities at all times, in the form of sleep, wakefulness and work, are presented. By comparing the bottom plot to the top plot, one can see the relation between the sleep drive and whether the nurse is sleeping or is awake. As mentioned previously, the only effect of the rosters on the fatigue model is the forced wakefulness that occurs when working shifts (and commuting before and after work).

Note that night shifts are defined to begin during the end of a day, so the night shifts in Fig. 1 during days 2 and 3 begin at hours 46 and 70. From the activity plot it is clear that the nurse only got a short period of sleep between night shifts, falling asleep around hour 67, before the nurse was forced to awaken 45 minutes prior to the second night shift beginning at hour 70.

In this work, the fatigue model is taken to be the best representation available of a nurse’s fatigue at any time, and the fatigue scores provided by the model are thus sometimes referred to as the *true fatigue* of a nurse. In Section 3.2 the fatigue model is approximated for incorporation in NRPs. It is referred to as the *rolling horizon approximation* or simply *approximation*.

### Biological variations

In this work we wish to take into account that fatigue develops differently for different individuals, as “knowledge of individual circadian phase in shift workers could identify times of impaired alertness and thereby inform individualized countermeasures for improving workplace safety, overall health, and wellbeing.” [69] The model presented in [49] has previously been used to gain insights into the physiological basis for inter-individual differences in circadian timing (see e.g. [49] [64, 72]) and the circadian response to simulated shift work (see e.g. [52, 70]). It is thus a good fit for introducing individual biological differences. We present our approach to modelling biological variations as a set of 9 biological profiles in Section 6.1.

### Approximating the fatigue model

The fatigue model is inherently non-linear, and incorporating it in an NRP is not trivial. When creating a parameter to represent fatigue in our NRPwF, time is discretised into days.

Evaluating the fatigue created by all possible rosters of realistic sizes is futile. The number of possible rosters is simply too large. For example, for a roster with 4 shifts and a planning period of 42 days, an upper bound on the number of possible rosters is approximately $1.93\times 10^{25}$. The number of practically feasible rosters would be lower, but for realistic sets of constraints, the number of rosters would still be huge.

To deal with this issue, we develop a rolling horizon approximation of the fatigue model, using explicit enumeration of all possible rosters of a given number of days $T^{h}$. The rosters of length $T^{h}$ are stored in a lookup table. This approach implicitly assumes there exists a finite number of days ($T^{h}$) shorter than the planning horizon of the full roster, that provides a useful approximation of the fatigue. When evaluating a time period $[t-T^{h}+1,t]$, this period is referred to as the *evaluation horizon*. The sequence of shifts worked during the evaluation horizon is referred to as the *evaluation pattern*. For every evaluation pattern we elicit the nurse’s maximum fatigue on day *t*. Clearly the longer the horizon, the better the estimate. The best estimate from the model is, of course, when the complete work history of the nurse is entered into it: in effect this is an infinite horizon.

The action of performing an evaluation of a full individual roster, thus obtaining the model’s best possible prediction of the fatigue scores (the true fatigue), is referred to as a *full roster evaluation* (*FRE*). The action of performing an evaluation of an individual roster using the rolling horizon approximation is referred to as a rolling horizon evaluation (*RHE*). *RHEs* can be performed for different evaluation horizons *t*, indicated through the notation $RHE_{t}$.

**Table 1:** Demonstration of how the rolling horizon approximation evaluates the different 3-day patterns that exist as parts of *IndR*. The rolling horizon approximation uses the information from the last day of the evaluation patterns, and save them to comprise the approximated fatigue scores for all days. Shift codes in bold represent the scores stored for each evaluation pattern

Day	-1	0	1	2	3	4	5	6	7
IndR			N	N	O	D	D	E	O
IndP1	O	O	**N**						
IndP2		O	N	**N**					
IndP3			N	N	**O**				
IndP4				N	O	**D**			
IndP5					O	D	**D**		
IndP6						D	D	**E**	
IndP7							D	E	**O**

In Table 1, we present an example of a $RHE_{3}$, i.e. the rolling horizon evaluation given a three-day evaluation horizon. The 7-day individual roster $IndR = \{N,N,O,D,D,E,O\}$ begins on day 1 and it is approximated using a 3-day rolling horizon approximation. The true fatigue is found by evaluating the full *IndR* and storing the fatigue scores each day, while the approximation evaluates the 7 different 3-day individual rosters $IndP1\ldots IndP7$ and storing the fatigue score obtained on the last day of each pattern, as demonstrated in Table 1. For days 1 and 2, we assume off-days before the beginning of *IndP*3, which do not affect the initial values of the fatigue model. As a consequence, the 3-day *RHE* results match the *FRE* results for days $1\ldots 3$, but from thereon differences may arise.

We noted that the fatigue model’s initial values reflect a well-rested individual with a stable circadian rhythm before introducing the *RHE*. However, in the case of *RHEs*, some evaluation patterns can follow a night shift (see e.g. *IndP*5 in Table 1). Because a night shift stretches into the following day and forces a state of wakefulness in the beginning of that day, we introduce an additional initial fatigue model state for all evaluation patterns that succeed a night shift - essentially this is the *RHE* approximation extended to include the initial night-shift.

### Testing the rolling horizon approximation

We run our *FRE* and our *RHE*s for different evaluation horizons on a collection of 30 real-life rosters of 42 days worked by anonymous nurses at the Austin hospital in Melbourne to evaluate the quality of our approximation. We perform our analysis with $T^{h} \in [3, \ldots ,7]$. This is because preliminary testing implies $T^{h} \le 2$ is insufficient, and $T^{h} \ge 8$ would imply generating a very large lookup-table. The large look-up table would be time consuming to generate and potentially increase the complexity of our NRP, depending on implementation. It should be noted, the realistic length of the look-up table depends on the number of possible shifts that exist in separate time slots for each nurse; a larger collection of shift times will entail exponentially larger look-up tables. The first $T^{h}$ days of the *RHE*s will naturally be identical to the *FRE*.^2^ Thus, we disregard the data for the first 7 days. This gives us 30 rosters of 35 days for 9 biological profiles. Every day, in each roster, for all biological profiles, we identify the fatigue scores^3^, and thus get 9450 data points to compare the *FRE* with each of the *RHEs*.

A difference in sleep drive of 0.10 mV is regarded as irrelevant by the developer of the model in [49], thus there is a good match if in most cases the errors between *FRE* and *RHEs* are less than this. To evaluate the full model (*FRE*) the algorithm solves a differential equation using the MATLAB ordinary differential equation solver “ode23”, see [63]. The 0.10 mV benchmark for magnitudes of errors necessitated a significant reduction in the tolerances of the differential equation solver, as compared to the default values. Our tests included several rosters in violation of the Safe Work Australia guidelines, which should thus be considered relatively tough.

To evaluate the quality of the rolling horizon approximations of different evaluation horizons, we want to compare each data point in the *FRE* with each data point in the *RHEs*, by quantifying the errors of the approximations. For every *RHE* of a given evaluation horizon, for each data point, we subtract the value provided by the *RHE* from the value provided by the *FRE* for the same data point. E.g., for a 3-day rolling horizon approximation we find the value of $FRE-RHE_{3}$ for all 9450 data points. We then sort the errors, and obtain percentiles to get an overview of how large the errors are. Negative values in a given percentile would imply the *RHEs* are larger than the *FRE* and vice versa.

**Table 2:** Results of subtracting values of *RHEs* of different evaluation horizons from the *FRE* of 30 real rosters. Values for all biological profiles are used. (The units in the fatigue model are millivolts)

Evaluation Horizon	1st perc.	5th perc.	10th perc.	90th perc.	95th perc.	99th perc.
$FRE - RHE_{3}$	-1.5024	-0.3074	-0.1169	0.0224	0.0895	0.7230
$FRE - RHE_{4}$	-1.3593	-0.3014	-0.0969	0.0144	0.0631	0.6078
$FRE - RHE_{5}$	-1.1767	-0.2859	-0.0879	0.0088	0.0454	0.5438
$FRE - RHE_{6}$	-1.0473	-0.2606	-0.0743	0.0058	0.0411	0.4636
$FRE - RHE_{7}$	-1.0355	-0.2019	-0.0551	0.0049	0.0389	0.4968

Results of the analysis are presented in Table 2. Firstly, we note that errors decrease for longer evaluation horizons, as expected. For all percentiles, the longer evaluation horizons have errors closer to 0 in Table 2. Secondly, it is notable how the magnitude of the errors are larger than the irrelevant magnitude 0.1 (less than -0.1 or more than 0.1) in roughly $7\%$ of cases. Furthermore, it is notable that approximations both over- and underestimate the fatigue of nurses regularly. Arguably positive differences, underestimating the fatigue, is a worse error in case the *RHE* is used for rostering.^4^ However the underestimate, at the $95^{th}$ percentile, is still less than the irrelevant magnitude, and even at the $99^{th}$ percentile for the larger horizons the deviation is below 0.5mV. This compares with fatigue levels that rise to values over 6mV even in the most alert-safe rosters, and well over 10mV in a normal roster.

To understand how errors occur, we consider one of the evaluated 42-day rosters, denoted $realroster_{1}$.

$$\begin{aligned}&realroster_{1}=\\&\{O,O,E,E,D,O,O,E,E,D,O,O,E,D,\\&D,E,D,D,O,O,E,E,D,D,O,O,O,O,\\&O,N,N,N,O,O,O,N,N,N,O,O,O,O\} \end{aligned}$$

**Fig. 2:**
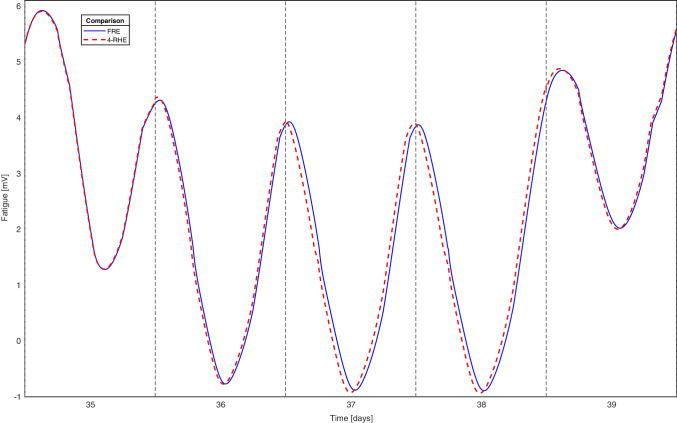
Excerpt of the fatigue on days 35 to 39 as $roster_{1}$ is evaluated through *FRE* and $RHE_{4}$. Notice a small error from the beginning of day 36, lasting throughout the days in the plot

During the first 30 days, the *FRE* and *RHE* are close to identical, with only irrelevant differences. On day 30, lasting into day 31, the nurse works the first of three consecutive night shifts, ending with a shift from late hours on day 32 until the morning on day 33. On days 34 and 35, the nurse is still recovering from this shift sequence, and fatigue is above rested levels. This leads to a small but visible error appearing few days later as presented in Fig. 2.

On day 36, the *FRE* has evaluated a long roster and has slightly different parameter values than in the fully rested state. If the nurse had some continuous off-days from day 36, the *FRE* would eventually fall back to fit the $RHE_{4}$ again, but as consecutive night shifts occur days 36, 37, and 38, the errors rather increase. As a result, errors marginally larger than the 0.1 threshold occur in several of the following days, although the shapes of the two graphs are very similar and intuitively imply high precision in the approximation.

However, in Table 2, some errors are far larger than those illustrated in Fig. 2. This is due to an additional effect, and we illustrate a particularly tough roster from the collection of real rosters to demonstrate it, $realroster_{2}$:

$$\begin{aligned}&realroster_{2}=\\&\{O,O,O,N,N,N,N,O,O,O,N,N,N,N,\\&O,D,O,N,N,N,O,O,O,O,N,N,N,N,\\&O,O,O,N,N,N,N,O,O,O,N,N,N,O\} \end{aligned}$$

**Fig. 3:**
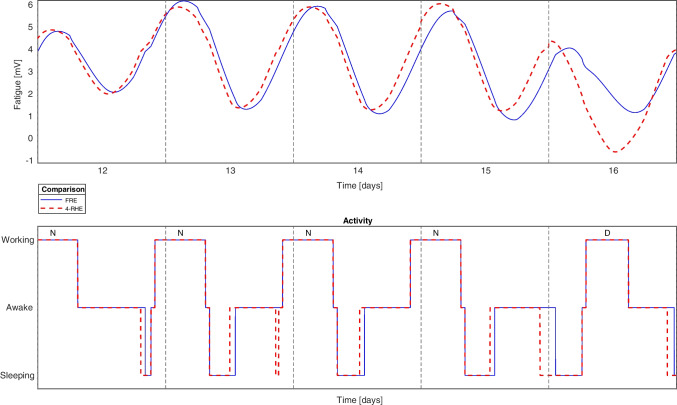
Excerpt of both the fatigue and the corresponding activity on days 12 to 16 as $roster_{2}$ is evaluated through *FRE* and $RHE_{4}$

In $realroster_{2}$, the consecutive night shifts on days 4 to 7 lead to a similar shift in the circadian rhythm as we observed in Fig. 2. However, when the second sequence of four consecutive night shifts occur, one can observe an interesting difference in the activity plot in Fig. 3. On day 13, the $RHE_{4}$ calculates that the nurse will have a short nap before going to work (notice the dip in the red dotted activity plot), while the *FRE* does not. From that day and onwards, the two graphs diverge consistently both in terms of maximum daily fatigue values and in the shapes of the two graphs.

The $RHE_{4}$ calculates a nap on day 13 because it just exceeds a threshold for specific values of brain activity inherent in the fatigue model (not just the sleep drive/fatigue value), while the *FRE* only comes close to that same threshold. In real life it is unclear whether the nurse would, in fact, sleep at this time or not. However the divergence between *FRE* and *RHE* shows that the two scenarios - staying awake or sleeping at this time - has significant knock-on effects for the nurse’s alertness.

The notion that minor errors at any point in time can lead to different activities and thus escalate into large differences, brings up an important consideration. In reality, the nurse might or might not have a nap prior to the night shift, depending on a variety of external factors such as noise, light, telephone interruptions etc. Consequently it could be *FRE* that diverges from reality and not *RHE*. In this work, however, we regard the *FRE* is the best model available for predicting real-life sleep patterns and treat it as a prediction of true fatigue values.

With this discussion in mind, for *RHE* we choose a 4-day horizon in our computational experiments. This gives a practical size of the NRPwF, accepting that whatever the horizon, some differences from *FRE* are likely to occur.

## The nurse rostering problem with fatigue

In this Section, we present a brief formal problem description in Section 4.1 and a model formulation in Section 4.3. As we use a mix of different techniques for implementation of our model, we provide a short explanation of key concepts in Constraint Programming before presenting the model according to Mixed-Integer Programming-tradition.

### Problem description

The following hard constraints are based on Safe Work Australia’s guide for managing the risk of fatigue at work [61]. Every day in the planning period, each nurse should be allocated either one work shift or an off-day. At least a minimum number of nurses must be assigned to work on each day, evening and night shift. The number of successive night shifts is restricted. After ending a night shift, or a sequence of consecutive night shifts, every nurse should have two consecutive nights without work. Nurses should not be assigned backward rotation, meaning that on the day after a shift, the next shift should be the same shift type, or a shift starting later. Restricting backward rotation thus ensures minimum rest times between shifts. There is also an upper limit to the number of consecutive days of work a nurse can have.

Nurses have a maximum number of hours they cannot exceed on average throughout the planning horizon, and for realism we also constrain the average minimum number of hours. Furthermore, there exists a maximum number of hours a nurse can work in any week. In our case, this can be regarded as a maximum number of weekly shifts, as all work shifts last 8.5 hours (see Section 3). Nurses are guaranteed to have a weekend off with a given frequency. A weekend off is defined as not working the night shift Friday, any shift Saturday, nor the day or evening shift Sunday. Two consecutive off-days should be ensured for each nurse with a reasonable frequency.

We formulate an objective function reflecting the discussions of fairness and patient safety in Section 2.1. We thus minimise the highest fatigue experienced by any nurse at any time in the planning period; the global maximum fatigue (GMF).

This objective function has some weaknesses. It can be brittle in the sense that it does not take into account any other fatigue scores than the very worst one. As a result, rosters could have arbitrarily high fatigue scores for other nurses and on other days, as long as they are below the GMF, without deteriorating the objective function value.

Furthermore, rostering is a multi-faceted task, and multiple different qualities are typically desired in most NRPs; both for theoretical and especially for practical problems. In comparison our objective function is very simplistic. However, this is done to highlight the effects of introducing our novel approach to modelling fatigue, and incorporating other qualities to rosters should be considered interesting themes of future research.

### Fatigue scores

We believe the most relevant value to represent a nurse’s fatigue throughout a day, both in terms of patient safety and nurse health, is the highest fatigue experienced during the 24 hours of that day.

Notice that high fatigue scores increase during forced wakefulness, when at work or commuting, and these are the times at which performance is crucial. Outside these hours we assume the nurse is able to sleep when fatigued, so the maximum fatigue score only occurs while working or commuting.

We base our modelling of the NRPwF on approximated *RHE* fatigue scores, which may differ from scores returned by the full *FRE* model, as discussed previously. However, we perform a full *FRE* evaluation on the rosters our system computes, and asses the effects of such errors afterwards.

Another issue affected by our objective function is fairness. It is quite typical in nurse rostering to model fairness as treating all nurses in the same way, e.g. restricting the difference in working tiring or unpopular shifts. However, we envisage an alternative perspective on fairness, where avoiding the highest fatigue levels for every nurse is more fair than treating everyone the same. We also argue it is more fair to patients to minimise fatigue levels of staff and to avoid huge differences in alertness among nurses. We thus believe minimising the maximum fatigue level is interesting and arguably can result in more fair rosters. It should be noted that the traditional scheduling rules are in place, which treat every nurse the same regardless of their biology. This limits how differently nurses can be scheduled, as the rules treat every nurse the same.

### Modelling the problem

The NRPwF is modelled in the MiniZinc language [46], which can map the model onto either MIP or CP solvers, or hybrids. While we choose to formulate the model according to MIP tradition in this work, some key CP concepts utilised in the algorithm should be explained briefly.

Unlike MIP, where constraints must be in linear form, a CP specification (model) can use more expressive built-in constraints (e.g. *not equal*, *append*, *alldifferent*, etc.), and even new constraints defined within the specification. This flexibility helps to simplify the specification, reflect the original problem definition, and is well suited for the problem presented in this paper.

In CP, a problem is defined by a set of variables, representing the choices to be made in reaching a solution; constraints, representing properties/requirements of the problem which must be satisfied in any solution; and the objective, whose value is to be optimised. Each variable can take a set of values, known as its *domain*, and each constraint involves a subset of these variables. In CP, a process called *filtering* is performed first where an appropriate resolution method is applied on each constraint to reduce the domains of its variables; i.e. the values of variables that violate the constraint are removed. When a domain of the variable is changed, it is beneficial to run through all constraints that contain this variable and see whether this change leads to new domain reductions. This process is called *propagation*. Iteratively, a variable is chosen and a value from its domain is assigned to it. The filtering and propagation process is triggered on each assignment. This sometimes leads to the removal of all the values of a variable resulting in a failed value assignment. In the event of a failure, the latest value assignment is reconsidered, called *backtracking*, and a new value is tried. The iterative value assignment, and backtracking process is called *search*. So, as defined in [58], CP is based on three strategies: filtering, propagation and search.

The choice of variables and constraints in a CP model can impact its efficiency. In particular, the use of sophisticated *global* constraints, which have specialised filtering algorithms, can enhance its performance. Therefore, when modelling our problem, we have used the ‘regular’ global constraint to capture requirements that apply to every sequence of rostered days or shifts; and the ‘cardinality’ constraint to enforce coverage for each shift in a roster.

Before presenting our model, we note that a symbol directory and a full model formulation are available in Appendix 1. In the NRPwF we assign nurses $n \in \mathcal {N}$ to shifts $s \in \mathcal {S}$. $\mathcal {S}$ consists of all the work shifts $\mathcal {S}^{W}$ and the off-shift $s^{O}$. The work shifts consist of day, evening and night shifts ($s \in \mathcal {S}^{W}= \{s^{D},s^{E},s^{N}\}$). They are allocated during all days in a defined planning period $t \in \mathcal {T}$. In this problem we assume all nurses have had a long period of off days before the beginning of this roster. For constraints stretching back in time to days prior to the defined set of days, we thus assume all nurses were assigned off-shifts $s^{O}$. That is, for all values of $t\le 0$, variables $y_{nst}$ and $z_{nt}$ should be interpreted as fixed and corresponding to off-shifts. As some restrictions apply specifically for weekends, a set of Sundays $\mathcal {T}^{S}$ is defined such that $\mathcal {T}^{S}=\{t \in \mathcal {T}|mod(t,7)=0\}$.

The model in the rest of this section, constraints Eq. 1 - Eq. 12, is formalised using MIP integer-linear inequations. In our implementation, they are actually modelled and implemented using CP global constraints, and solved using filtering, propagation and search.

#### Coverage

Nurses are assigned through binary variables $y_{nst} \in \{0,1\}$. $y_{nst}=1$ if nurse *n* works shift *s* on day *t*; 0 otherwise.

1 $$\begin{aligned} \sum _{n \in \mathcal {N}} y_{nst} \ge \underline{P}^{C}_{s},\;&s \in \mathcal {S}^{W}, t \in \mathcal {T} \end{aligned}$$

Constraints Eq. 1 ensure coverage, by enforcing that required staffing levels $\underline{P}^{C}_{s}$ must be respected for all work shifts *s* on all days.

#### Short-term rest

To ensure sufficient rest, different shift transitions and limitations on work patterns are not allowed.

2 $$\begin{aligned} \sum _{s \in \mathcal {S}} y_{nst} =1,\;&n \in \mathcal {N}, t \in \mathcal {T} \end{aligned}$$

3 $$\begin{aligned} \sum _{d = t-\overline{P}^{CN}}^{t}y_{ns^{N}d} \le \overline{P}^{CN} ,\;&n \in \mathcal {N}, t \in \mathcal {T}\end{aligned}$$

4 $$\begin{aligned} y_{ns^{N}(t-1)} + y_{ns^{D}t} + y_{ns^{E}t} \le 1,\;&n \in \mathcal {N}, t \in \mathcal {T} \end{aligned}$$

5 $$\begin{aligned} y_{ns^{E}(t-1)} + y_{ns^{D}t} \le 1,\;&n \in \mathcal {N}, t \in \mathcal {T} \end{aligned}$$

6 $$\begin{aligned} y_{ns^{N}(t-2)} + \sum _{s \in \{s^{D},s^{E},s^{O}\}}y_{ns(t-1)}&\nonumber \\ + y_{ns^{N}t} \le 2,\;&n \in \mathcal {N}, t \in \mathcal {T} \end{aligned}$$

7 $$\begin{aligned} \sum _{s \in \mathcal {S}^{W}}\sum _{\tau = t-\overline{P}^{CD}}^{t}y_{ns \tau } \le \overline{P}^{CD},\;&n \in \mathcal {N}, t \in \mathcal {T} \end{aligned}$$

Constraints Eq. 2 enforce that exactly one shift is assigned to each nurse per day. Constraints Eq. 3 ensure no nurse works more than $\overline{P}^{CN}$ consecutive nights. Constraints Eq. 4 and Eq. 5 make sure that backward rotation is not possible, thus securing a minimum period of rest between shifts for all nurses. Constraints Eq. 6 state that it is not possible to work a night shift followed by an off day and then another night shift. This implies that after ending a sequence of consecutive night shifts, the nurse will not be working during the night in any of the two following days. Constraints Eq. 7 set the maximum number of consecutive work days to $\overline{P}^{CD}$.

#### Long-term rest

To ensure two consecutive days of rest, a new variable is introduced. $z_{nt}$ is a binary auxiliary variable used to indicate if a nurse is allocated any work shifts during a period of two consecutive days ending on day *t*. Due to nurse preferences, the two-day period considered includes the night shift on day $t-2$ rather than the night shift on day *t*. This especially affects weekends, as $z_{nt}=0$, $t \in \mathcal {T}^{S}$, implies nurse *n* has the nights off on Friday and Saturday when they have the weekend off.

8 $$\begin{aligned} \underline{H} \le \sum _{s \in \mathcal {S}}\sum _{t \in \mathcal {T}}{P^{H}_{s}} y_{nst} \le \overline{H} ,\;&n \in \mathcal {N}\end{aligned}$$

9 $$\begin{aligned} \sum _{s \in \mathcal {S}^{W}}\sum _{\tau = t-6}^{t}{P^{H}_{s}}y_{ns\tau }\le \overline{H}^{W},\;&n \in \mathcal {N}, t \in \mathcal {T}^{S} \end{aligned}$$

10 $$\begin{aligned} 2z_{nt}-y_{ns^{N}(t-2)}-\sum _{s \in \mathcal {S}^{W}}y_{ns(t-1)}&\nonumber \\ -y_{ns^{D}t}-y_{ns^{E}t}\ge 0,&n \in \mathcal {N},\;t \in \mathcal {T} \end{aligned}$$

11 $$\begin{aligned} \sum _{\tau =0}^{\overline{P}^{CW}} z_{n(t-7\tau )} \le \overline{P}^{CW},\;&n \in \mathcal {N}, t \in \mathcal {T}^{S} \end{aligned}$$

12 $$\begin{aligned} \sum _{\tau = t-\overline{P}^{Z}}^{t}z_{n\tau } \le \overline{P}^{Z},\;&n \in \mathcal {N}, t \in \mathcal {T} \end{aligned}$$

Constraints Eq. 8 restrict the hours worked by each nurse to be in the interval [$\underline{H},\overline{H}$]. The length of shift s is denoted ${P^{H}_{s}}$. Constraints Eq. 9 restrict working more than $\overline{H}^{W}$ hours every week. Constraints Eq. 10 ensure $z_{nt}$ indicates work during a two-day period. Due to the short-term rest constraints in Section 4.3.2, the big *M*-value 2 is sufficient in constraints Eq. 10. Constraints Eq. 11 ensure that no nurse works $\overline{P}^{CW}$ consecutive weekends. Furthermore, every nurse should have two consecutive days off at least once every $\overline{P}^{Z}$ days, as instructed through constraints Eq. 12.

#### Objective function

The variable $f_{nt}$ represents the fatigue score of nurse $n \in \mathcal {N}$ on day $t \in \mathcal {T}$. The value of $f_{nt}$ is retrieved from a lookup table where there are multiple additional inputs. The biological profile $b \in \mathcal {B}$ of nurse *n*, information on whether the nurse worked a night shift the day prior to the evaluation pattern, and the evaluation pattern itself are all relevant inputs for the value of $f_{nt}$. However, we use the simple $f_{nt}$ syntax here, and present a more detailed version in Appendix 1. Here, auxiliary variable $f^{GM}$ is introduced to represent the global maximum fatigue score, and is assigned the correct value due to constraints Eq. 13. The objective function is presented in constraint Eq. 14.

13 $$\begin{aligned} f^{GM} - f_{nt}\ge 0\;n \in \mathcal {N}, t \in \mathcal {T} \end{aligned}$$

14 $$\begin{aligned} \text {Minimise } f^{GM} \end{aligned}$$

## The solution method

Our solution method is presented and discussed here, firstly through an introduction to the overall idea and then through a more detailed description in Section 5.1.

The general idea of the solution method can be summarized as follows:

1. Find initial solution running the NRP without the fatigue model
2. Run Large Neighbourhood Search iteratively to reduce the approximated global maximum fatigue
  - When possible, directly minimise the approximated GMF
  - When necessary, reduce the number of cases where an identical approximated GMF value is observed in the roster
3. Evaluate final roster to identify true GMF

The above steps correspond to the main solution algorithm. For a more complete review of it, pseudo-code is provided in Algorithm 1 in Appendix 1.

When the true GMF is identified, we consider post-processing if the true GMF is notably larger than the approximated GMF. For a more complete review of the post-processing procedure, see Algorithm 2 in Appendix 1.

### Description

We first use a MIP solver to find a feasible solution when the fatigue model is not included in the model; our current best solution. From this point on, in every iteration we have a current best solution available, with a current roster parameter denoted $y^{*}_{nst}$ and a current global maximum fatigue parameter denoted $f^{GM*}$. We also denote the current 4-day rolling horizon approximated fatigue parameter of nurse *n* on day *t* $f^{*}_{nt}$, and introduce the current individual maximum fatigue parameter of nurse *n* $f^{IM*}_{n}$, defined as the highest fatigue experienced by nurse *n* in the planning period $f^{IM*}_{n} = max_{t \in \mathcal {T}}(f^{*}_{nt})$.

To reduce complexity when performing iterations, we fix the roster $y_{nst}$ to be identical to the roster in the current best solution $y^{*}_{nst}$, except for some specifically chosen combinations of nurses *n* and days *t* denoted by the neighbourhood parameter $N_{nt}$. If $N_{nt}$ takes the value 0 then $y_{nst}$ is fixed to the value of $y^{*}_{nst}$, otherwise (if it takes the value 1), then $y_{nst}$ can be allocated a new value

In every iteration of our algorithm, we create a new roster $y_{nst}$ with new approximated fatigue scores $f_{nt}$ resulting in a new GMF $f^{GM}$. In every iteration, the algorithm either reduces the GMF ($f^{GM}<f^{GM*}$), finds a new solution with unchanged GMF and fewer occurrences of the GMF ($f^{GM}=f^{GM*}\bigcap (sum_{n\in \mathcal {N}, t\in \mathcal {T}}(f_{nt}=f^{GM*})<sum_{n\in \mathcal {N}, t\in \mathcal {T}}(f^{*}_{nt}=f^{GM*}))$), or is not able to find a better solution and keeps the current best ($y_{nst}=y^{*}_{nst}$). Attempting to reduce the GMF is the standard approach, while attempting to reduce occurrences of the GMF is done when symmetry or unsuccessful previous attempts indicate this is more promising.

**Fig. 4:**
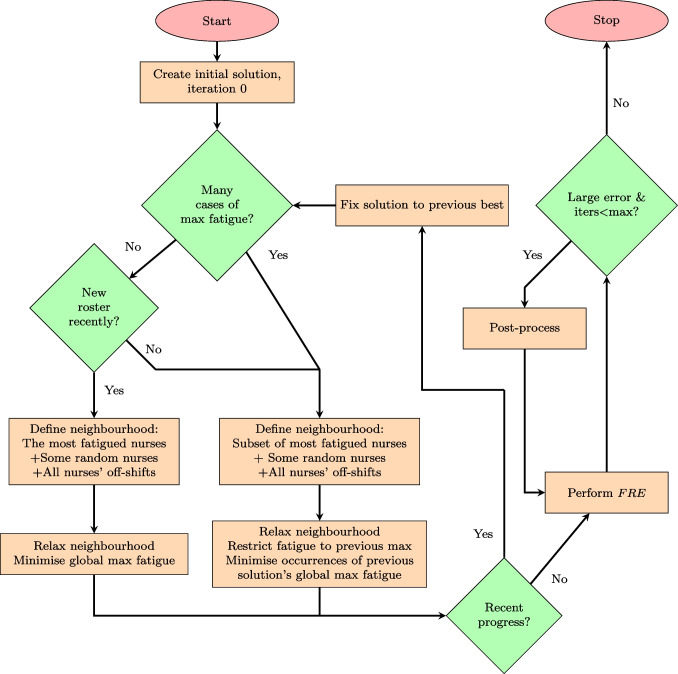
Flow chart of algorithm

When we are no longer able to improve the solution, we perform a full roster evaluation of it, and if errors have relevant magnitude, we follow the process in Section 5.1.3. We provide a conceptual illustration of the algorithm in Fig. 4 with a brief description of each step.

#### Minimising GMF

As seen from Fig. 4, the algorithm firstly creates an initial solution, identifying $y^{*}_{nst}$ and $f^{GM*}$. Assume there are *not* many occurrences of the $f^{GM*}$ and that a new solution *has* been found recently, the standard optimisation approach of *minimising the GMF* is implemented. This means we implement the objective function presented in constraint Eq. 14 in Section 4. All combinations of nurses and days described below constitute the neighbourhood when performing the standard optimisation approach:

1. The full individual schedules of a set of nurses $\mathcal {N}^{F}$ experiencing the GMF ($N_{nt} = 1, \ n \in \mathcal {N}^{F}, t \in \mathcal {T}$, provided that $n \in \mathcal {N}^{F}| f^{IM*}_{n}=f^{GM*}, n \in \mathcal {N}$)
2. The full individual schedules of a set of $n^{R}$ random nurses $\mathcal {N}^{R}$ ($N_{nt} = 1, \ n \in \mathcal {N}^{R}, t \in \mathcal {T}$, provided that $\mathcal {N}^{R}=rand(\mathcal {N}-\mathcal {N}^{F},n^{R})$
3. The off-shifts of all nurses in the roster ($N_{nt}=1, \ n\in \mathcal {N}, t \in \mathcal {T}|y_{ns^{O}t}=1$)

We fix the roster $y_{nst}$of any iteration to be equal to the current best roster $y^{*}_{nst}$, except for the defined neighbourhood where $N_{nt}=1$, as below:

15 $$\begin{aligned} y_{nst}=y^{*}_{nst},\;n \in \mathcal {N}, s \in \mathcal {S}, t \in \mathcal {T}| N_{nt}=0 \end{aligned}$$

#### Minimising occurrences of the global maximum fatigue

Assume the response to the question of recent progress in Fig. 4 is “yes”, and another iteration is performed. If there *are* many occurrences of nurses experiencing the GMF ($f_{nt}=f^{GM*}$ for more than some few $n \in \mathcal {N}, t \in \mathcal {T}$) and/or our algorithm has *not* been able to produce a new solution in recent iterations (we consider ourselves stuck in a local optima). In this case, the neighbourhood is defined in the same way as for the standard optimisation approach, with the notable exception that if there are more than $n^{F}$ nurses in $\mathcal {N}^{F}$, the set is redefined as a randomly drawn subset of maximum $n^{F}$ of the nurses experiencing the GMF. Furthermore, we *minimise the occurrences of the GMF*. This also entails restricting the maximum fatigue score of all nurses to the current best BMF. While the nonlinearities are handled by the CP solver in our algorithm, we still provide a MIP linearisation for intuitive understanding. Let the binary variable $f^{Occ}_{nt} \in \{0,1\}$ be equal to 1 if nurse *n* experiences the GMF on day *t*, 0 else.

16 $$\begin{aligned}&f_{nt} \le f^{GM*} ,\;n \in \mathcal {N}, t \in \mathcal {T}\end{aligned}$$

17 $$\begin{aligned}&f^{Occ}_{nt} - f_{nt} + f^{GM*} > 0 ,\;n \in \mathcal {N}, t \in \mathcal {T} \end{aligned}$$

18 $$\begin{aligned} \text {Minimise } \sum _{n \in \mathcal {N}}\sum _{t \in \mathcal {T}} f^{Occ}_{nt} \end{aligned}$$

Constraints Eq. 16 ensure that no nurse is assigned a fatigue score that is higher than the GMF of the previous iteration, while constraints Eq. 17 state that $f^{Occ}_{nt}$ must take a value higher than 0 if $f_{nt} = f^{GM*}$, but can be 0 otherwise. Minimising $f^{Occ}_{nt}$ in constraint Eq. 18 thus entails minimising the number of occurrences of the GMF.

#### Ensuring *RHE* is close to *FRE*

Assume, after some iterations of the algorithm in Fig. 4, that there has been no *recent progress* (the evaluation of this question is discussed in more detail in Section 6.2) A *FRE* is performed to unveil the true fatigue of the best solution, before the GMF of the *FRE* is compared to $f^{GM*}$.

If the true GMF turns out higher than the $f^{GM*}$, with a margin larger than the 0.10mV threshold of relevance, we perform post-processing and repeat the *FRE* on the new roster produced in post-processing. We assume extras (casuals / interim nurses) can step in on some limited number of shifts when necessary, as is common in real-life. In our solution method, that means we can substitute a tiring shift in our roster with an off-day. Post-processing is thus done by removing the shift prior to the true GMF identified in the *FRE*. This is repeated until the true GMF is below $f^{GM*}+0.10$mV or a maximum number of post-processing iterations is reached. The post-processing is discussed further in Section 6.2.1, and pseudocode is available in Algorithm 2 in Appendix 1.

## Computational study

The algorithm is run using Python3.6.8 to define neighbourhoods and calls MiniZinc 2.3.2 using the built-in MIP-solver gurobi8.1.1 to find the initial solution and the CP-solver Chuffed 0.10.4 [15] to search within the given neighbourhoods. Matlab R2018 is called to create the lookup-table of approximated fatigue values and to perform the *FRE*. Computational experiments are run using an HP EliteBook 820 G3 with the specifications below:

$$\begin{aligned} CPU:\;&Intel\;Core\;i7-6500U\;CPU\;@\;2.50GHz - 2\;cores\\ RAM:\;&16Gb \end{aligned}$$

### Instances

When performing computational studies, we would ideally use real instances. However, as collecting information about individual biology would be both complicated and controversial, we create biological profiles $b \in \mathcal {B}$ by making changes to two parameters in the fatigue model that represent common differences in biology related to sleep [49]. These two parameters represent the average sleep-time and the chronotype of a human.

The average-sleep-times parameter is by default calibrated for a person with a normal chronotype, which is $\approx$ 7 hours sleep when fully rested beforehand. According to the developer of the model of [49], 5 and 9 hours are realistic variations within the adult population in real-life. To identify the right parameter values for the sleep times, we thus vary the one parameter typically reflecting this in the adult population to get the right hours of sleep (see constant offset $D_{0}$ in [49]), while all other parameters are left at their default values. To get a meaningful number of these nurses represented, we draw biological profiles for nurses with a 10% chance of having a short sleep time ($\approx$ 5 hours) or a long sleep time ($\approx$ 9 hours), leaving an 80% probability of the most common value ($\approx$ 7 hours). The chronotype parameter is by default set to its standard value representing the most common “day-time chronotype”. We similarly provide chronotype parameters that are somewhat uncommon, but within realistic variations in the adult population, to create “morning-type” and “evening-type” biological profiles (see intrinsic period $\tau _{c}$ in [49]). We assume these two parameters are not correlated, and thus produce the 9 different biological profiles in Table 3.

**Table 3:** Illustration of the probability of drawing different biological profiles for the nurses in our instances. The index-value of each of the biological profiles is given in parenthesis

		Average sleep time		
		Short $\approx$ 5	Normal $\approx$ 7	Long $\approx$ 9
Chronotypes	Probabilities	0.1	0.8	0.1
Morning-type	0.1	0.01 (5)	0.08 (4)	0.01 (6)
Day-type	0.8	0.08 (2)	0.64 (1)	0.08 (3)
Evening-type	0.1	0.01 (8)	0.08 (7)	0.01 (9)

In Table 3, the probability of drawing each profile is given depending on the average sleep time and the chronotype of any given nurse. Clearly this very coarse grouping of biological profiles does not come close to capturing the real-world variations of biology affecting sleep. However, the profiles facilitate analyses of the effects of some common differences in biology among nurses. Furthermore, as these parameters represent two aspects of sleep that would be possible to unveil using e.g. a survey, our profiles represent a realistic and pragmatic approach to including biology in real-life rostering.

Furthermore, deciding on the number of nurses relative to minimum staffing levels, as ensured by coverage constraints, is not trivial. We have used real-life 12-week rosters from the Intensive Care Unit (ICU) at the Alfred in Melbourne, Australia as a basis for the minimum staffing parameter. This ICU is an ideal starting point for creating instances that are realistic while ensuring that biological profiles are the only differences between nurses. Firstly, the ICU is the largest in the state of Victoria in Australia [68], making it scalable despite the skill-mix variations in real-life rostering problems. Secondly, the activity at the ICU is inherently interminable, and as a result shift work is planned around the clock.

In the real-life data, several nurses work part-time. This counteracts the desired homogeneousness of our nurses, but by calculating the ratios between full-time equivalents and day, evening and night shifts, we calculate conservative estimates of the minimum coverage requirements. These are 7, 5, and 5 nurses for day shifts, evening shifts, and night shifts respectively, given a total staff of 30 full-time nurses, and correspond to the parameter $\underline{P}^{C}_{s}, s\in \mathcal {S}^{W}$ in the model in Section 4. Other data and parameter values are retrieved from the Safe Work Australia guidelines [61], and can be found in Appendix 1. As a result of this approach to creating instances, the only difference between instances is the number of nurses with each biological profile, unless stated otherwise. We generate 20 instances and analyse them in the following sections.

### Minimising the global maximum fatigue scores

**Fig. 5:**
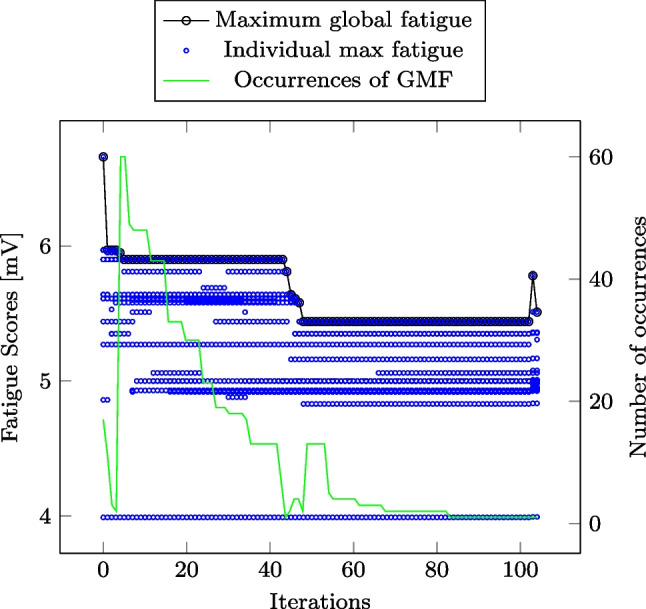
Figure demonstrating change in the value, and the number of occurrences, of the global max fatigue for Instance 1. Iteration 0 is the initial feasible solution, while Iterations 1 to 101 are based on the approximated fatigue scores. Iteration 102 is the true global maximum fatigue score and Iteration 103 is the result of post-processing

In Fig. 5, the minimisation of the global maximum fatigue score of Instance 1 is illustrated. Black circles with the black lines striking through represent the GMF ($f^{MG*}$) in each iteration. Blue circles represent one or more nurse’s individual maximum fatigue score ($f^{IM*}_{n}$) in each iteration (some circles are hidden behind each other). The green line represents the number of occurrences of the current GMF in each iteration ($\sum _{n \in \mathcal {N}}\sum _{t \in \mathcal {T}}f^{Occ}$).

In Fig. 5, Iteration 0 simply produces any feasible solution to the NRPwF; a roster that respects all scheduling rules defined in the Safe Work Australia guidelines. Note that these guidelines correspond to the standard constraints in nurse rostering literature, meaning that the GMF of the roster obtained in iteration 0 corresponds to what one would expect to see in a typical NRP paper. As our model iterates to reduce the GMF, we thus find solutions that increasingly outperform typical rosters in terms of the single highest fatigue score.

During the first iterations, the global maximum fatigue clearly decreases, before stabilising at 5.90. Had we only performed 40 iterations on this instance, and not noted the change in number of occurrences of the GMF, we would likely conclude that the algorithm seemed to converge. However, from the green line in Fig. 5 we can see that while the global maximum fatigue score remains 5.90 during iterations 5 to 43, the number of occurrences of the 5.90-score gradually decreases from 60.

From this point, the global maximum fatigue continues to decrease from iteration 43 to 48, converging to 5.44. For this value of the GMF, however, the number of occurrences also remains unchanged, and we can thus be more confident we have found a high-quality solution. These results illustrate that rather than determining an exact number of iterations for all instances, we should terminate our algorithm when we have reason to believe the maximum global fatigue score has converged, i.e. when our algorithm has no progress in a reasonable number of iterations. We set this limit to 20 iterations without either finding a new GMF or reducing the number of occurrences of the GMF.

#### Errors and post-processing

In Fig. 5, there is a spike in the fatigue score in iteration 103. Iteration 103 represents the *FRE* performed after termination of our algorithm. From the large difference in fatigue score in iteration 102 and iteration 103, there is clearly at least one case of significantly higher true GMF than estimated in iteration 102. To counteract this, we use extras, as mentioned briefly in Section 5.

In reality, it is common in most hospitals to have access to some number of extras that can cover for staff when necessary. We assume that a ward of 30 full-time nurses has access to extras that can cover one shift per week on average, i.e. up to six shifts can be covered by extras in our planning period. When the true GMF is 0.10mV, or more, higher than the approximated GMF, the last work day prior to the true GMF is replaced with an off-shift for the nurse experiencing the GMF. If the process is performed six times and the global maximum fatigue is still more than 0.10mV larger than the approximated global maximum fatigue score, we assume that we have exhausted the ward’s budget for extras on single shifts, and accept that the fatigue is higher than implied by the approximation. Note that in introducing the post-processing procedure, we implicitly allow for violations of the minimum total work hours ensured by Constraints Eq. 8. Pseudocode for the post-processing procedure is presented in Algorithm 2 in Appendix 1.

In Fig. 5, the last iteration, iteration 104, is the result of using an extra worker for one single shift. In the example of Instance 1, the approximated global maximum fatigue value was 5.44, the true evaluation was 5.78, and after post-processing (adding one off-shift) the true fatigue value became 5.51.

**Table 4:** The table presents global maximum fatigue scores for 20 instances. All values are given in milliVolts

	Initial	Values based on $RHE_{4}$							Values based on *FRE*				
Instance	Iter 0	Iter 10	Iter 20	Iter 40	Iter 60	Iter 80	Iter 100	Last	True	Error	1 extra	2 extras	3 extras
1	6.66	5.90	5.90	5.90	5.44	5.44	5.44	5.44	5.78	0.34	5.51		
2	6.66	5.90	5.90	5.44	5.35	5.35		5.35	5.35	0.00			
3	6.66	5.90	5.90	5.90	5.90	5.90		5.90	5.92	0.02			
4	6.66	5.90	5.90	5.44	5.35	5.35		5.35	5.35	0.00			
5	6.66	5.90	5.90	5.44	5.44	5.44		5.44	5.47	0.03			
6	6.66	5.90	5.90	5.90	5.90			5.90	6.19	0.29	5.91		
7	6.66	5.90	5.44	5.44	5.35	5.35	5.35	5.35	6.13	0.78	5.36		
8	6.66	5.90	5.90	5.90	5.90	5.66	5.44	5.44	5.50	0.06			
9	6.66	5.90	5.90	5.90	5.44	5.44	5.35	5.35	5.36	0.01			
10	6.66	5.90	5.90	5.90	5.53	5.44	5.44	5.44	5.54	0.10	5.44		
11	6.66	5.90	5.90	5.90	5.90	5.90		5.90	5.94	0.04			
12	6.66	5.90	5.90	5.59	5.35	5.35	5.35	5.35	5.36	0.01			
13	6.66	5.90	5.90	5.90				5.90	6.06	0.16	6.05	6.01	5.96
14	6.66	5.97	5.90	5.90	5.90	5.90	5.90	5.90	5.97	0.07			
15	6.66	5.90	5.81	5.44	5.35	5.35		5.35	5.89	0.54	5.7	5.69	5.36
16	6.66	5.90	5.90					5.90	6.06	0.16	6.01	5.96	
17	6.56	5.90	5.90	5.44	5.44	5.44	5.44	5.44	5.51	0.07			
18	6.66	5.90	5.90	5.90	5.90	5.35	5.35	5.35	5.36	0.01			
19	6.66	5.90	5.90	5.90	5.90	5.90		5.90	6.21	0.31	6.06	5.95	
20	6.66	5.90	5.90					5.90	6.08	0.18	5.98		

In the column of Iteration 0 in Table 4, the global maximum fatigue scores of the initial feasible roster is provided. The subsequent columns up to the column denoted “Last” present the global maximum fatigue based on the approximated fatigue scores. Later columns all include the true fatigue scores provided through *FREs* and errors found by comparing the *FREs* with the approximated fatigue score in the latest iteration of our algorithm. If errors are greater or equal to the threshold of relevance, 0.10mV, extras are used to cover single shifts, and new *FRE*-values are provided in subsequent columns.

Immediately we notice the stark difference between GMF in rosters in Iteration 0 and the column denoted “Last”. As all feasible solutions to the NRPwF must respect the Safe Work Australia guidelines, one might not expect the potential for reducing the GMF was very large to begin with. However, results in Table 4 underscore that the algorithm is able to reduce the approximated GMF vastly by explicitly minimising it (1.06mV on average for all instances).

From results in Table 4, it is clear that post-processing is necessary in 9 of the 20 instances to reduce the error to an acceptable magnitude. In five cases one extra shift is sufficient, in two cases we need two extras, and in two cases we need three extras. The post-processing technique seems effective, as evidenced by the results in Table 4. Notably, all errors are positive (*FRE*-scores are larger than $RHE_{4}$-scores), which can seem surprising given results in Table 2. However, the global maximum fatigue scores obtained after *FRE* are not necessarily the same nurses and shifts that are estimated to have the maximum global fatigue by the $RHE_{4}$. With 30 nurses and 42 days in a roster, there could potentially be large true fatigue scores in seemingly arbitrary parts of the final roster, as became clear when analyzing errors in Section 3.3. With this in mind, it makes sense that errors tend to be positive.

Results in Table 5 demonstrate that focusing only on the approximated values from our lookup-table (in practise this means focusing on shorter shift patterns) does not guarantee against producing tiring schedules for some nurses (see e.g. Instance 7), but it proves to be a highly useful proxy. The longer shift patterns are considered, the better the proxy, as implied by results in Section 3.3. Furthermore, managers can ensure a high-quality roster if they combine this approach with a full evaluation of rosters after they are created and also use extras for single shifts. Our results suggest this number can be small, and in many cases 0.

#### Roster insights

The large plateaus in Fig. 5 stand out as an interesting characteristic, which provides some insights to the structure of the NRPwF when minimising the global maximum fatigue. Note that 5.90 is the approximated fatigue score for a nurse of biological profile 1 working the shift pattern {O,N,N,N} or the pattern {D,N,N,N}. In other words, to reduce the objective function value, all such patterns for nurses of biological profile 1 must be removed without introducing an even higher fatigue score somewhere in the roster. This is an example of a pattern that can occur frequently, as the probability of a nurse having biological profile 1 is 64%, and three consecutive nights is the maximum number of nights.

However, as the algorithm has removed high fatigue scores from the roster, this has affected the number of different shifts worked by nurses of different biological parameters. In Table 5 we present some key information on the average number of different shifts worked by nurses of each biological profile. 30 nurses working 42 days and a minimum of 7 day shifts, 5 evening shifts, and 5 night shifts per day, implies each nurse should work a minimum of 9.8 day shifts and 7 evening and night shifts each, as seen in the last row in Table 5.

**Table 5:** Roster statistics for each of the 9 biological profiles in the 20 instances. The first three columns identify each biological profile’s sleep time and chronotype, while columns 4-7 give average numbers of different shift types allocated

Biological	Sleep	Chrono-	Avg.	Avg.	Avg.	Avg. Nr.
profile	time	type	Day	Evening	Night	triple night
1	Normal	Day	10.41	8.21	7.10	0.10
2	Short	Day	6.94	6.10	12.51	0.55
3	Long	Day	13.33	9.82	2.59	0.00
4	Normal	Morning	10.11	7.18	8.44	0.49
5	Short	Morning	5.00	6.00	14.75	2.50
6	Long	Morning	12.83	1.17	11.83	1.33
7	Normal	Evening	10.79	6.49	8.51	0.59
8	Short	Evening	14.56	2.22	9.22	1.67
9	Long	Evening	16.40	8.20	1.40	0.00
Avg. Min.			9.8	7	7	

It is clear from Table 5 that nurses of different biological profiles are assigned very different numbers of different shifts. Nurses of the most typical biological profile 1 work on average 10.41 day shifts, 8.21 evening shifts, and 7.10 night shifts. They thus work approximately their share of each shift type. Nurses of biological profile 1 work on average 0.10 of the tiring triple night-patterns. The low number of triple night-patterns is notable, and make sense seeing that most instances in Table 4 have no approximated fatigue scores of 5.90.

Nurses of all the nine biological profiles have little negative impact on their sleep from working day shifts, and it is thus natural to compare profiles by looking at the number of night shifts and evening shifts they are able to perform without producing high fatigue scores. If we analyse the values in Table 5, we can see that the sleep times tend to affect the number of shifts worked during hours the nurse would otherwise sleep. Nurses that have short sleep times, i.e. nurses of biological profiles 2 (12.51 nights), 5 (14.75 nights), and 8 (9.22 nights), work more night shifts than the minimum requirement per nurse.

On the other hand, nurses with long sleep times work few night shifts on average (profile 3 works 2.59 and profile 9 works 1.40) with the exception of the morning chronotype profile 6 (11.83 nights). The case of profile 6 is interesting, because it contradicts a notion of a straight-forward relation between length of sleep times and the frequency of night shifts. However, we can see that the number of evening shifts worked by nurses of biological profile 6 is 1.17. This could mean that for nurses with a morning chronotype, it is more problematic to work evening shifts and easier to work night shifts than for nurses of other chronotypes. Intuitively this makes sense, as morning type sleepers go to bed early and as a result this can make evening shifts more challenging than for other chronotypes. Also, with most other biological profiles clearly favouring evening shifts over night shifts, it is practical not to assign evening shifts to nurses of profile 6 from a combinatorial perspective.

It seems that when minimising the global maximum fatigue, we must take special notice of the needs of nurses with long sleep times and customize the rosters for them. This entails treating nurses differently in order for them to be similarily fatigue when they are the most tired, possibly denouncing the simplified fairness rules typically presented in rostering literature, where e.g. nurses are assigned the same number of the unpopular night shifts. The customization could both entail ensuring sufficient off-days and rest times, but it is also notable that the chronotype of a nurse seems to decide which shifts are most disadvantageous to the nurses.

### The effects of increased staff levels on maximum fatigue levels

To analyze the effect of staff size on the global maximum fatigue score in a roster, we take Instances 1-20 solved in Section 6.2 and add one or two full-time nurses of biological profile 1 to evaluate the effects. The instances with extra full-time nurses are simply referred to with +1 or +2 in subscripts, e.g. Instance $1_{+1}$ and $FRE_{+1}$. Results based on *FRE*s are presented in Table 6.

**Table 6:** True fatigue scores of running our algorithm on Instances 1-20 with none, one and two full-time extra nurses added to the staff. Average values in the last row

Instance	$FRE^{pp}$	$FRE^{pp}_{+1}$	$FRE^{pp}_{+2}$	$FRE^{pp}_{+3}$	$FRE^{pp}_{+1}$-$FRE^{pp}_{+2}$
1	5.51	5.36	5.30	5.19	0.32
2	5.35	5.35	5.35	4.94	0.41
3	5.92	5.80	5.36	5.35	0.57
4	5.35	5.35	5.31	5.22	0.13
5	5.47	5.36	5.35	5.35	0.12
6	5.91	5.38	5.19	5.15	0.76
7	5.36	5.36	5.36	5.36	0.00
8	5.50	5.46	5.47	5.34	0.16
9	5.36	5.37	5.24	4.9	0.46
10	5.44	5.44	5.30	4.92	0.52
11	5.94	5.90	5.36	5.34	0.60
12	5.36	5.35	5.36	5.35	0.01
13	5.96	5.36	5.36	5.35	0.61
14	5.97	5.82	5.80	5.35	0.62
15	5.36	5.40	5.36	5.39	-0.03
16	5.96	5.49	5.47	5.44	0.52
17	5.51	5.43	5.47	5.35	0.16
18	5.36	5.35	5.36	5.36	0.00
19	5.95	5.37	5.35	5.35	0.60
20	5.98	5.97	5.59	5.43	0.55
Average	5.63	5.48	5.39	5.27	0.35

In Table 6 we present true fatigue scores for all 20 instances after post-processing.^5^

There are some instances where the global maximum fatigue scores are reduced greatly in Table 6, while there are others that have no or very little improvement. From the average values in the last row, we can see a slightly larger average improvement from adding the first nurse in column $FRE^{pp}_{+1}$ compared to $FRE^{pp}$, than comparing the two later columns with their priors ($FRE^{pp}_{+1}$ to $FRE^{pp}_{+2}$ and $FRE^{pp}_{+2}$ to $FRE^{pp}_{+3}$), but average differences in GMF from adding a nurse are generally small. It is interesting to compare the second column $FRE^{pp}$ containing true fatigue scores after post-processing for the original instances with the last column, as we do in $FRE^{pp}$ -$FRE^{pp}_{+3}$, where the total improvement in GMF from adding three nurses to the staff is presented.

It is clear that instances with the highest GMF in the original instances $FRE^{pp}$ have the largest improvements. That is, Instances 3, 6, 11, 13, 14, 16, 19, and 20 all have improvements of 0.50 or more, and they all had $FRE^{pp}$-values in the region of 5.90-6.00 in Table 6. These improvements have quite clearly come as a direct result of being able to remove triple night-patterns for the nurses of a normal biological profile and in some cases adding extra off-shifts to compensate for errors in our 4-day rolling horizon approximation. On the other hand, of the 7 instances that had $FRE^{pp}$-values in the range 5.30-5.40 (Instance 2, 4, 7, 9, 12, 15, and 18) in the original instance, only three had an improvement of relevant magnitude.

Despite errors occurring when performing *FRE*s, there is only one case of increased objective function values when calculating the $FRE^{PP}_{3}$-$FRE^{PP}$ (Instance 15). For this instance, the results of approximated GMFs was unchanged when three nurses were added to the group of staff, and the error when performing the *FRE* happened to be larger for the $FRE^{PP}_{3}$ than the $FRE^{PP}$. However, this error is well within the 0.10 threshold of relevance. Results in Table 6 thus seem realistic.

The above mentioned results highlight two interesting insights. Firstly, and perhaps unsurprising to practicing nurses, increased staff levels enable less tiring rosters. Managers should note that avoiding the most undesirable shorter patterns tend to reduce nurse fatigue. Secondly, when biological profiles are as coarsely divided as in our experiments, the effects of removing every occurrence of a short and tiring pattern becomes important. When adding an additional nurse means the last nurse of biological profile 1 no longer has to work any triple night shifts, the GMF is typically reduced by a lot. If adding the additional nurse only reduces the occurrences of triple night shifts among nurses with biological profile 1, the GMF is unchanged or changed within the threshold of relevance. However, in reality, every individual’s biology will differ to some extent, and if this information was truly available and incorporated in the fatigue model, there would likely be small reductions in the GMF in cases where our model only shows a reduction in occurrences of the GMF. It is therefore reasonable to look at the average values of reduction of the GMF in Table 6 to estimate the effect of adding one additional nurse to the staff. Thus, the average values of 0.14, 0.11 and 0.10 mV decrease in GMF per added staff are likely reasonably close to the actual decrease one can expect from adding a full-time nurse to a ward of 30 full-time nurses. Simply put, the reduction in fatigue by adding an additional nurse is small, but not irrelevant.

### The value of knowing each individual’s biotype

While utilizing sleep models in rostering is in itself uncommon at most real-life hospital wards, the introduction of different biological profiles is especially novel. To evaluate the impact of it, we run our algorithm minimising the global fatigue score for a set of 30 nurses that all have the normal biological profile 1. When the final roster is produced, we perform new *FRE*s on the final roster, this time applying other biological profiles to all nurses. That is, all nurses are evaluated assuming biological profile 2, before all nurses are evaluated assuming biological profile 3, etc. Essentially we test how well we can minimise global fatigue scores without taking into account individual biology.

**Table 7:** Key fatigue score statistics for the same roster where all nurses are assumed to have the biological profile in the leftmost column. The roster was produced by our algorithm minimising the global maximum fatigue score for 30 nurses, all with biological profile 1

Biological	Sleep	Chrono-	Global maximum
profiles	time	type	fatigue score
1	Normal	Day	6.00
2	Short	Day	5.64
3	Long	Day	6.65
4	Normal	Morning	5.62
5	Short	Morning	5.05
6	Long	Morning	6.38
7	Normal	Evening	6.09
8	Short	Evening	5.26
9	Long	Evening	7.00
Average			5.96

In Table 7 the global maximum fatigue scores are provided. Comparing the values in this column across all biological profiles, we see that the standard profile 1 has a global maximum fatigue score of 6.00, which is near the average maximum global fatigue score across all biological profiles of 5.96.

Table 7 provides some pointers to which profiles contribute to increasing and reducing the fatigue. The profiles with global maximum fatigue scores higher than biological profile 1 are profiles 3 (6.65), 6 (6.38) and most notably 9 (7.00). Nurses with these three biological profiles have in common their long sleep time. This indicates that the long sleep time is a key characteristic of the nurses that are easily fatigued, which corresponds to results in Section 6.2.2. In those experiments, long time sleepers were spared the most tiring shifts. In this case, such individual customization was not made, and the global maximum fatigue scores of long time sleepers is far higher than in any of the rosters produced in Section 6.2 as a result. Similarly, short time sleepers tend to have lower fatigue scores than biological profile 1 had in Table 7, with scores 5.64 for profile 2, 5.05 for profile 5, and 5.26 for profile 8.

We note that the morning chronotype nurses all have a lower global maximum fatigue score than the day and evening chronotype nurses with the same sleep times (profile 4 has lower global maximum fatigue score than profiles 1 and 7, etc.) This is interesting, especially knowing that rosters were created to minimise the global maximum fatigue of nurses with a day chronotype. This indicates that it is advantageous for a nurse to have a morning chronotype over a day chronotype, although results would be dependant on shift definitions and commuting. The difference between day and evening chronotypes is more unclear, as global maximum fatigue scores are 6.00, 5.64, and 5.82 for day chronotypes and 6.09, 5.26, and 7.00 for evening chronotypes in Table 7.

By revisiting results in Section 6.2 of running our 20 instances in Table 4, an interesting realisation occurs, that the fatigue score of the normal biological profile 1 in Table 7 is in fact quite poor. The approximated GMF for the case of 30 nurses with biological profile 1 is 5.90 (triple night pattern) and the true GMF is 6.00.^6^ The poor results for 30 nurses of the normal biological profile 1 imply that variations in biological profiles are beneficial when minimising the GMF. With the insights acquired in Section 6.2.2 in mind, it seems nurses with different chronotypes act as complementary resources at the ward, an interesting notion for those looking to recruit new shift workers.^7^

To date practical studies on the impact of medical staff rosters on patient outcomes, have only explored the more obvious causes of fatigue such as long shifts and high working hours per week. For example [66] records that “nurses working more than 50 h/week showed the highest adverse nurse outcome scores.”

We look forward to the deployment of alert-safe rosters which will allow their calculated fatigue scores to be correlated with reported patient impacts.

## Conclusions and future work

First and foremost, this work demonstrates that prospective use of advanced sleep models in nurse rostering is realistic. The work is generalisable to other industries, and is a first step towards reaching the next frontier of individualised fatigue mitigation.

We have presented and formalised the Nurse Rostering Problem with Fatigue by approximating and incorporating an advanced sleep model in a general NRP. An algorithm combining Mixed-Integer Programming and Constraint Programming to form a Large Neighbourhood Search was introduced. The algorithm created high-quality rosters minimising the global maximum fatigue for all nurses. Instances were created as 6-week rosters using real-life data. We further demonstrated the use of a post-processing technique in cases where approximation errors are larger than a threshold of relevance.

### Technical outcomes

Nurse rostering is a time-consuming task, and poor rostering choices can easily result in fatigue and medical errors. This paper described how a fatigue model can be successfully integrated with a nurse rostering model and solved to a practical scale (30 nurses over 6 weeks) using a hybrid algorithm. Current systems implement rules, such as the guidelines in [61], to avoid fatiguing rosters. The results show that, compared with a roster which merely implements such a generic set of rules, levels of fatigue can be reduced by more than 1.00*mV* with an integrated fatigue model in the rostering system.

### Managerial insights

In practice, managers should be aware of the potential benefits for nurse health and patient safety, and innovative health care institutions should consider pilot projects with real-life implementation.

Among other results, our research demonstrates two closely linked insights:

1. Minimising the global maximum fatigue for nurses of different biological profiles entails assigning them different numbers of shifts during evening and night time
2. Without customisation to individual nurses’ biology, we cannot expect to create rosters that limit, as well as possible, the highest fatigue levels

In a practical setting this means that managers must treat nurses differently in order to minimise the global maximum fatigue. This entails grappling with an idea of what fairness is in rostering. While it is easy and tempting to treat every nurse exactly the same irrespective of their reaction to working round the clock, this does not suffice if managers wish to create rosters that focus on nurse health and patient safety.

Furthermore, our results support the intuitive notion that biological parameters linked to sleep time affect the fatigue experienced from shift work. That is, nurses who are rested and uninterrupted and sleep approximately 5 hours are more resilient to shift work than those who sleep 7 or 9 hours. Furthermore, the fatigue experienced from working at different hours seem to be affected by the chronotype of a nurse, and results from minimising the global maximum fatigue of nurses demonstrate that while day and evening chronotype nurses should not be assigned a lot of night shifts, the evening chronotype nurses should not be assigned too many evening shifts. Furthermore, our results indicate that nurses’ different chronotypes can prove complementary when creating rosters.

Our research demonstrates how minimally increasing the staff levels makes it possible to decrease the global maximum fatigue levels. Our results indicate that the average of global maximum fatigue scores decrease by a small but significant magnitude for each additional nurse, assuming the additional nurse has a normal biology in terms of sleep.

We have not been able to prove the optimality of the solutions we have obtained, due to the vast complexity of the problem. To guarantee the validity of managerial results, optimal solutions would be preferable. Nonetheless, we do not believe the current algorithm contains any biases that could affect the managerial results.

### Future work

Real-life implementation and evaluation of the impact on nurse health and patient safety would be very useful. It is not clear what the impact of our proposed model would have on a multitude of different real-life rostering aspects. What effect will it have on work culture when planners specifically focus on equity? Could this rather radical change in the understanding of fairness imply conflict among staff? And what legal aspects should be considered when using fatigue tools for planning? We invite researchers to investigate such related topics.

Most real NRPs are in reality multi-objective problems, and it would be interesting to see the effects of combining our proposed minimisation of the worst fatigue scores with other objectives such as individual preferences, other fairness measures, and personnel costs. Introducing other objectives to the NRPwF would change the problem significantly, and likely imply the need for a new solution method, and so these topics of future research are closely intertwined. The current post-processing method is realistic, but simple. It would be interesting to see work where alternative strategies to simply removing shifts and assigning an off-day are considered.

The general approach for incorporating the sleep model, where the approximation is created through a look-up table, is likely useful in OR within other areas of application. Furthermore, as sleep models are improved, research on rostering using sleep models should be updated and improved. Noting the vast impact of variations in two of the most common biological profiles, it would be very interesting to see the impact of more refined biology in nurses. From a rostering perspective, it would be particularly interesting if future sleep models were able to take into account how other factors such as individuals’ social life etc. affects sleep patterns. There are examples of attempts at this, see e.g. [51]. For many nurses, work-life balance includes a preference towards following the circadian rhythm of the rest of society, to enable daily chores and meeting others with a more standard work schedule.

## Figures and Tables

**Algorithm 1:**
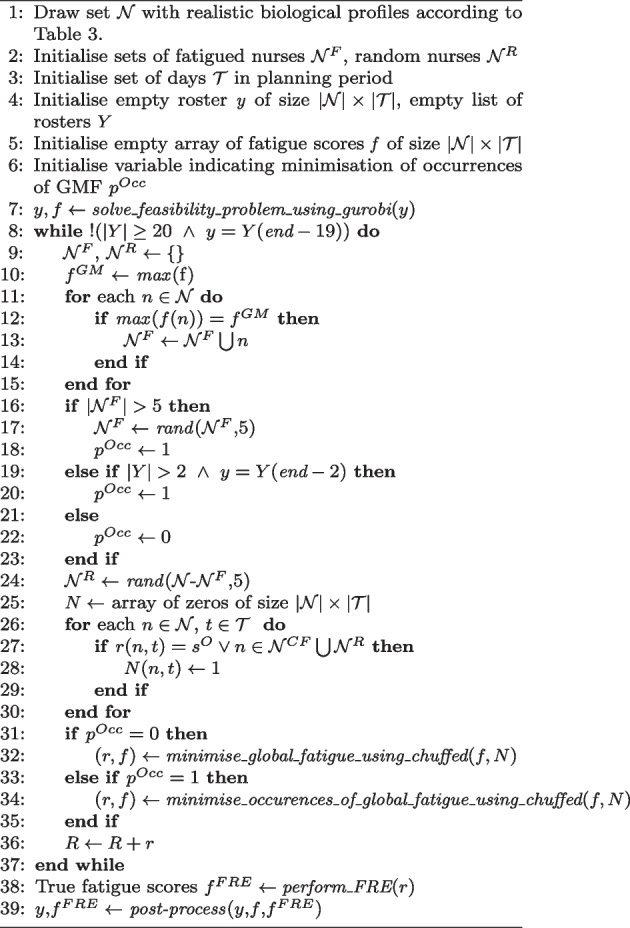
**Solving the Nurse Rostering Problem with Fatigue.**

**Algorithm 2:**
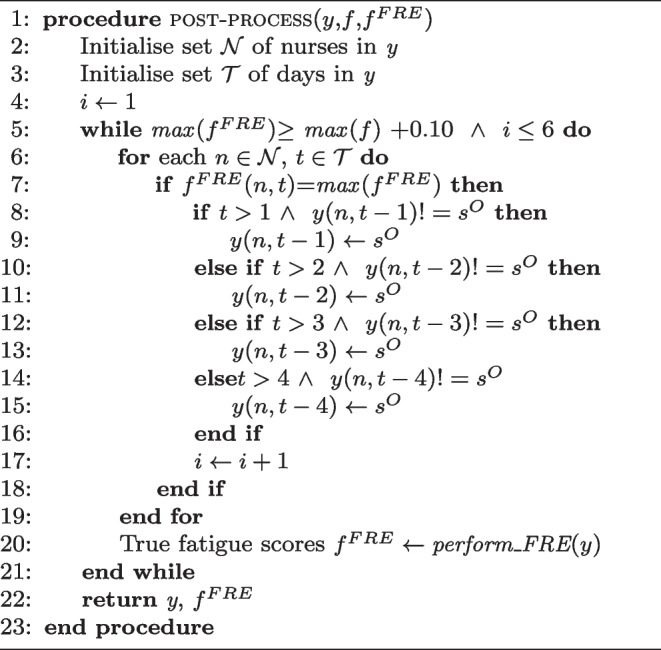
**Post-processing function.**

## A.1 Indices

**Table:**

Symbol	Description
*n*	Nurse
*s*	Shift
$s^{D}$	Day shift
$s^{E}$	Evening shift
$s^{N}$	Night shift
$s^{O}$	Off-shift
$t,\tau$	Day
*b*	Biological profile
*w*	Indication of night shift one day prior

## A.2 Sets

**Table:**

Symbol	Description	Range
$\mathcal {N}$	Nurses	1$\dots$ 30
$\mathcal {T}$	Days	1$\dots$ 42
$\mathcal {T}^{S}$	Sundays	7,14$\dots$ 42
$\mathcal {S}$	Shifts	$\{s^{D}, s^{E}, s^{N}, s^{O}\}$
$\mathcal {S}^{W}$	Work shifts	$\{s^{D}, s^{E}, s^{N}\}$

## A.3 Parameters

**Table:**

Symbol	Description	Value
$\underline{P}^{C}_{s^{D}}$	Minimum staff coverage of work shift day	7
$\underline{P}^{C}_{s^{E}}$	Minimum staff coverage of work shift day	5
$\underline{P}^{C}_{s^{N}}$	Minimum staff coverage of work shift day	5
$\underline{H}$	Minimum total work hours	210
$\overline{H}$	Maximum total work hours	228
$P^{H}_{s^{D}}$	Length of day shift [hours]	8.5
$P^{H}_{s^{E}}$	Length of evening shift [hours]	8.5
$P^{H}_{s^{N}}$	Length of night shift [hours]	8.5
$\overline{P}^{CN}$	Maximum number of consecutive night shifts	3
$\overline{P}^{CD}$	Maximum number of consecutive work days	6
$\overline{H}$	Maximum number of weekly work hours	50
$\overline{P}^{CW}$	Maximum number of consecutive work weekends	2
$\overline{P}^{CW}$	Maximum number of days between two off-days	10

## A.4 Decision variables

$y_{nst} \in \{0,1\}$ is a binary variable determining if shift $s \in \mathcal {S}^{W}$ is worked by nurse $n \in \mathcal {N}$ on day $t \in \mathcal {T}$. $y_{nst}$ constitutes the roster in the NRPwF.

$z_{nt} \in \{0,1\}$ is a binary auxiliary variable indicating if nurse $n \in \mathcal {N}$ works during a two-day period ending on a day $t \in \mathcal {T}$. $f_{n}^{Max}$ is a continuous variable equal to the maximum fatigue score value of nurse $n \in \mathcal {N}$ for the entire planning period.

## A.5.1 Coverage

19 $$\begin{aligned} \sum _{n \in \mathcal {N}} y_{nst} \ge \underline{P}^{C}_{s},\;&t \in \mathcal {T}, s \in \mathcal {S}^{W} \end{aligned}$$

## A.5.2 Short-term rest

20 $$\begin{aligned} \sum _{s \in \mathcal {S}} y_{nst} =1,\;&n \in \mathcal {N}, t \in \mathcal {T}\end{aligned}$$

21 $$\begin{aligned} \sum _{\tau = t-\overline{P}^{CN}}^{t}y_{ns^{N}\tau } \le \overline{P}^{CN} ,\;&n \in \mathcal {N}, t \in \mathcal {T}\end{aligned}$$

22 $$\begin{aligned} y_{ns^{N}(t-1)} + y_{ns^{D}t} + y_{ns^{E}t} \le 1,\;&n \in \mathcal {N}, t \in \mathcal {T} \end{aligned}$$

23 $$\begin{aligned} y_{ns^{E}(t-1)} + y_{ns^{D}t} \le 1,\;&n \in \mathcal {N}, t \in \mathcal {T} \end{aligned}$$

24 $$\begin{aligned} y_{ns^{N}(t-2)} + y_{ns^{O}(t-1)} + y_{ns^{N}t} \le 2,\;&n \in \mathcal {N}, t \in \mathcal {T} \end{aligned}$$

25 $$\begin{aligned} \sum _{s \in \mathcal {S}^{W}}\sum _{\tau = t-\overline{P}^{CD}}^{t}y_{ns \tau } \le \overline{P}^{CD},\;&n \in \mathcal {N}, t \in \mathcal {T} \end{aligned}$$

## A.5.3 Long-term rest

26 $$\begin{aligned} \underline{H} \le \sum _{s \in \mathcal {S}}\sum _{t \in \mathcal {T}}{P^{H}_{s}} y_{nst} \le \overline{H} ,\;&n \in \mathcal {N}\end{aligned}$$

27 $$\begin{aligned} \sum _{s \in \mathcal {S}^{W}}\sum _{\tau = t-6}^{t}{P^{H}_{s}}y_{ns\tau }\le \overline{H}^{W},\;&n \in \mathcal {N}, t \in \mathcal {T}^{S} \end{aligned}$$

28 $$\begin{aligned} 2z_{nt}-y_{ns^{N}(t-2)}-\sum _{s \in \mathcal {S}^{W}}y_{ns(t-1)}&\nonumber \\ -y_{ns^{D}t}-y_{ns^{E}t}\ge 0,\;&n \in \mathcal {N}, t \in \mathcal {T} \end{aligned}$$

29 $$\begin{aligned} \sum _{\tau =0}^{\overline{P}^{CW}} z_{n(t-7\tau )} \le \overline{P}^{CW},\;&n \in \mathcal {N}, t \in \mathcal {T}^{S} \end{aligned}$$

30 $$\begin{aligned} \sum _{\tau = t-\overline{P}^{Z}}^{t}z_{n\tau } \le \overline{P}^{Z},\;&n \in \mathcal {N}, t \in \mathcal {T} \end{aligned}$$

## A.5.4 Objective function

31 $$\begin{aligned} f^{GM} - f_{nt}\ge 0\;&n \in \mathcal {N}, t \in \mathcal {T} \end{aligned}$$

32 $$\begin{aligned} \text {Minimise } f^{GM} \end{aligned}$$

## A.5.5 Alternative objective function used in cases of many cases of GMF or no recent progress

$f^{Occ}_{nt} \in \{0,1\}$ is a binary variable equal to 1 if nurse *n* experiences the GMF on day *t*, 0 else. $f^{GM*}$ is the global maximum fatigue parameter from the previous iteration.

33 $$\begin{aligned} f_{nt} \le f^{GM*} ,\;&n \in \mathcal {N}, t \in \mathcal {T}\end{aligned}$$

34 $$\begin{aligned} f^{Occ}_{nt} - f_{nt} + f^{GM*} > 0 ,\;&n \in \mathcal {N}, t \in \mathcal {T} \end{aligned}$$

35 $$\begin{aligned} \text {Minimise } \sum _{n \in \mathcal {N}}\sum _{t \in \mathcal {T}} f^{Occ}_{nt} \end{aligned}$$

## A.6 The relation between the fatigue score variable and the lookup-table

Assume the lookup-table is described by parameter $P^{Score}_{bs_{1}s_{2}s_{3}s_{4}w}$, where index *b* is the biological profile, indices $s_{1}$, $s_{2}$, $s_{3}$, and $s_{4}$ are the shifts assigned on days $t-3$, $t-2$, $t-1$, and *t* of the evaluation pattern, and the index *w* is 1 if nurse *n* worked a night shift prior to the evaluation pattern, 0 else. The constraints below ensure a linear relation between variables $f_{nt}$ and the lookup-table of fatigue scores. We also introduce the binary auxiliary variable $p_{ns_{1}s_{2}s_{3}s_{4}wt}$, indicating if nurse *n* works the pattern indicated by the *s*-indices prior to a night shift or not on day *t*. Furthermore, we introduce the sets $\mathcal {B}$ consisting of all biological profiles and the sets $\mathcal {N}^{B}_{b}$ consisting of all nurses of biological profile *b*.

36 $$\begin{aligned}&p_{ns_{1}s_{2}s_{3}s_{4}1t} -y_{ns^{N}t-4}- y_{ns_{1}t-3} \nonumber \\&- y_{ns_{2}t-2}- y_{ns_{3}t-1} - y_{ns_{4}t} \le -4,\nonumber \\&n \in \mathcal {N}, s_{1}, s_{2}, s_{3}, s_{4} \in \mathcal {S}, t\in \mathcal {T}\end{aligned}$$

37 $$\begin{aligned}&p_{ns_{1}s_{2}s_{3}s_{4}0t} -\sum _{s \in \mathcal {S}\backslash s^{N}}y_{nst-4} - y_{ns_{1}t-3} \nonumber \\&- y_{ns_{2}t-2} - y_{ns_{3}t-1} - y_{ns_{4}t}\le -4,\nonumber \\&n \in \mathcal {N}, s_{1}, s_{2}, s_{3}, s_{4} \in \mathcal {S}, t\in \mathcal {T}\end{aligned}$$

38 $$\begin{aligned}&\sum _{s_{1} \in \mathcal {S}} \sum _{s_{2} \in \mathcal {S}} \sum _{s_{3} \in \mathcal {S}} \sum _{s_{4} \in \mathcal {S}} \sum _{w=0}^{1} p_{ns_{1}s_{2}s_{3}s_{4}0t}=1, \quad n\in \mathcal {N}, t\in \mathcal {T}\end{aligned}$$

39 $$\begin{aligned}&f_{nt} - P^{Score}_{bs_{1}s_{2}s_{3}s_{4}w}p_{ns_{1}s_{2}s_{3}s_{4}wt}\ge 0,\nonumber \\&b \in \mathcal {B}, n \in \mathcal {N}^{B}_{b}, s_{1}, s_{2}, s_{3}, s_{4} \in \mathcal {S}, w \in \{0,1\}, t\in \mathcal {T} \end{aligned}$$

Constraints Eq. 36 ensure that the variable $p_{ns_{1}s_{2}s_{3}s_{4}1t}$ can only have the value 1 if nurse *n* works the evaluation pattern defined by the s-indices if it occurs subsequent to a night shift, while constraints Eq. 37 ensure the same relation if the evaluation pattern occurs subsequent to a different shift than the night shift. Constraints Eq. 38 ensure that the variable $p_{ns_{1}s_{2}s_{3}s_{4}wt}$ can only be equal to 1 once for every combination of nurses and days, ensuring that the variable $p_{ns_{1}s_{2}s_{3}s_{4}wt}$ becomes an indicator of the evaluation pattern and prior night shift for nurse *n* on day *t*. Constraints Eq. 39 ensure the variable $f_{nt}$ cannot be lower than the approximated fatigue score of nurse *n* in the lookup-table if the nurse works the evaluation pattern and prior night shift defined by *s*-indices and *w* on any day *t*. Algorithm 1  Algorithm 2

## Footnotes

1 The neuro-physiological model of sleep drive is a voltage across certain neurons, measured in millivolts

2 Except for possible errors due to the use of numerical methods in the differential equation solver

3 Specified in Section 4.2 below.

4 The *RHE* seeks to approximate the full *FRE* evaluation of a roster as closely as possible. If the *RHE* is used for rostering, and predicts a lower level of fatigue than the *FRE*, then it is likely to be an underestimate of the actual fatigue due to the roster. In this case a roster predicted to be alert-safe by *RHE*, could (with low probability) cause fatigue and consequently result in medical errors. If, on the other hand, the *RHE* predicts a higher level of fatigue than the *FRE*, then it is likely that the real fatigue is overestimated. A roster based on this *RHE* might be non-optimal in terms of staff level, but would be alert-safe, and not result in fatigue causing medical errors.

5 To evaluate the effect of adding full-time nurses to instances, we could look at both the *FRE*-values without post-processing and the *FRE*-values with post-processing. However, ignoring post-processing entails assuming managers would ignore surprisingly high fatigue scores, and we believe this is unrealistic. We thus compare the cases with post-processing, and use pp in superscript to mark this (e.g. $FRE^{pp}_{+1}$), but note that the number of extra shifts introduced in post-processing varies, from 0 (most common) up to six (the maximum number of added off-shifts).

6 The error is just below 0.10 in this roster, and thus no post-processing occurred. Some rosters were post-processed in Table 4, making a direct comparison of the GMF between rosters imperfect.

7 An interesting parallel to the notion of using complementary chronotypes in scheduling exists in [74], where the reason for differences in chronotypes are theorised from an evolutionary perspective. [74] argue that humans likely evolved to co-sleep as families or even tribes, and that different chronotypes would reduce the time they were collectively vulnerable, thus enhancing safety.
